# Evaluation of Diabetes Care parameters in capillary blood collected with a novel sampling device

**DOI:** 10.1016/j.plabm.2019.e00135

**Published:** 2019-09-19

**Authors:** M.J.A.J. Huijskens, R. Castel, H.J. Vermeer, F.M. Verheijen

**Affiliations:** a Department of Clinical Chemistry, Albert Schweitzer Hospital, Dordrecht, the Netherlands; b Department of Clinical Chemistry, Result Laboratorium C.V., Dordrecht, the Netherlands

**Keywords:** Hem-Col, Diabetes care parameters, Finger prick, Venepuncture, Method comparison, Time and temperature stability, CI, confidence interval, CV, coefficient of variation, G3P, glycerol-3-phosphate, EDTA, Ethylenediaminetetraacetic acid, HbA1c, Haemoglobin fraction A1c, HDL, high density lipoprotein, LDL, low density lipoprotein, LiHep, lithium heparin, LoA, limit of agreement, LoQ, limit of quantification

## Abstract

The aim of this study was to determine whether the Hem-Col method of obtaining and storing blood is an acceptable alternative to venepuncture for measuring Diabetes Care parameters. ***Design and methods***: Hem-Col is a novel blood collection device that is designed to collect capillary blood drawn with a finger prick. Hem-Col is a microtube containing an anticoagulant and a preservation buffer to enhance analyte stability in whole blood. The Diabetes Care parameters cholesterol, creatinine, HbA1c, high density lipoprotein (HDL) cholesterol, low density lipoprotein (LDL) cholesterol, and triglycerides were measured both in blood/plasma collected via Hem-Col and blood/plasma collected with venepuncture. The results were compared to assess the agreement between the two methods. ***Results***: HbA1c shows agreement after storage for up to 120 hours at temperatures ranging from 4 to 37 °C. Cholesterol, HDL cholesterol, LDL cholesterol, triglycerides and creatinine can be measured after 120 hours of storage in Hem-Col buffer, if high temperatures are avoided, and with the use of correction factors or adaptations to reported reference intervals. ***Conclusion***: Hem-Col is suitable for the measurement of HbA1c after storage for up to 120 hours at temperatures ranging from 4 to 37 °C. Cholesterol, creatinine, HDL cholesterol, LDL cholesterol and triglycerides can be measured after 120 hours of storage in Hem-Col buffer, if high temperatures are avoided. Further studies are required to determine whether Hem-Col can replace the venepuncture for the Diabetes Care parameters.

## Highlights

• Diabetes Care parameters show acceptable agreement with the use of correction factors.
• Diabetes Care parameters can be measured after storage for 120 hours in Hem-Col.
• Further studies are required to determine if Hem-Col can replace the venepuncture.

## 1 Introduction

Self-management in healthcare is becoming increasingly important, especially for chronic care patients. Self-management and patient empowerment can lead to cost reduction, improved patient independence, and better healthcare outcomes [1,2].

Patients with Diabetes Mellitus comprise a significant portion of chronic care patients, and diabetes prevalence is increasing worldwide [3]. Averse health outcomes, like kidney impairment and peripheral neuropathy, can be partly explained by ineffective or suboptimal disease management [4]. Streamlining and simplifying disease management could help to improve health outcomes within this patient group.

Chronic care patients undergo regular doctor’s visits and blood draws for laboratory testing to manage their chronic disease. These requirements are inconvenient, intrusive on their personal lives and coincide with high healthcare costs [1]. By using a finger prick instead of a venepuncture, patients could draw blood by themselves at their own convenience, without visiting the hospital, general practitioner or phlebotomy service resulting in less impact on their daily life. Moreover, blood collection via finger prick is minimally invasive, collects a small volume of blood and is relatively inexpensive.

In addition, laboratories put great effort in pre-analytic tracts to secure sample quality, since most analytes are sensitive to time and temperature in the currently available tubes [5,6]. The time between blood collection and the separation of the plasma or serum from the cells by centrifugation is a critical step in the pre-analytic process. The Clinical and Laboratory Standards Institute (CLSI) recommends the centrifugation of tubes within two hours after blood collection [7]. This poses a challenge to laboratories if phlebotomy is performed at remote locations from the laboratory, where qualified phlebotomists are available at specific locations for a limited amount of time. Therefore, an elaborate logistics network needs to be maintained to guarantee that all samples are centrifuged and handled properly to ensure their quality. Thus, both patients and laboratories would benefit from a self-sampling blood device that can be used by healthcare providers at their practice or patients at home.

The short stability of analytes in whole blood is a major bottleneck in the development of alternatives to venepuncture. Point of care devices could overcome this, but the cost aspects, training of non-laboratory staff, and test quality will be concerns when implemented at large scale. By improving the stability of analytes in whole blood for extended periods of time, e.g. to 5 days, blood could be collected at any place and time, resulting in a step forward in patient empowerment and reduction of healthcare costs.

In this study we investigated the novel blood collection device Hem-Col®, which is designed to collect capillary blood drawn with a finger prick. Hem-Col is a microtube containing an anticoagulant and a preservation buffer to enhance analyte stability in whole blood.

The aim of this study was to determine whether the Hem-Col method of obtaining and storing blood is an acceptable alternative to venepuncture for measuring the Diabetes Care parameters cholesterol, creatinine, HbA1c, high density lipoprotein (HDL), low density lipoprotein (LDL) and triglycerides. We describe the comparison of finger prick via Hem-Col versus venepuncture for testing of these parameters under different time and temperature conditions.

## 2 Materials and methods

### 2.1 Patient material

Random patients requiring blood collection agreed to have both capillary blood via Hem-Col and venous blood by regular venepuncture collected during one visit. Written informed consent was obtained from all patients. Blood collections were performed by selected experienced phlebotomists. Capillary blood was collected using a BD Microtainer® Contact-Activated Lancet (Franklin Lakes, New Jersey, USA), after disinfecting the finger to be used. Approximately 4–5 drops (200 μl) of blood were collected per tube. Venous blood was collected in Lithium Heparin and Na_2_EDTA tubes (BD, Franklin lakes, New Jersey, USA). Tubes were subsequently centrifuged (5 min at 1500 g) at indicated time points and analysed.

### 2.2 Hem-Col device

**Fig. 1:**
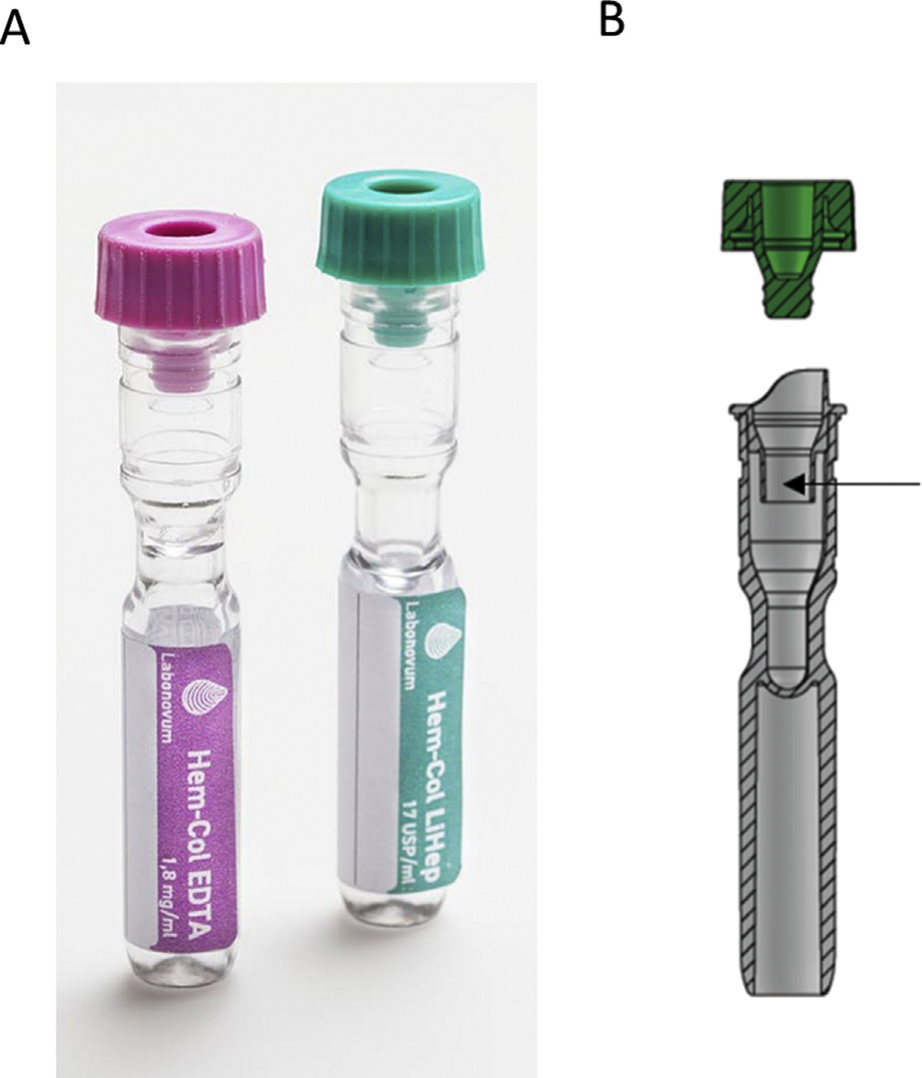
**Hem-Col Device.** A: EDTA and LiHep Hem-Col Devices. B: Schematic cross section of LiHep Hem-Col Device. The arrow indicates the liquid barrier.

The Hem-Col device containing either 17 USP/ml lithium heparin or 1.8 mg/ml EDTA dissolved in 150 μl preservation fluid (Hem-Col, Labonovum, Limmen, the Netherlands) was used, see Fig. 1. Hem-Col tubes have the size of regular blood collection tubes (13 × 75 mm) and are made of polyethylene, with a pierceable cap made of thermoplastic elastomers. All tubes contain a liquid barrier®, the inner part serves as a liquid barrier by preventing loss of Hem-Col conservation fluid and the outer part is used as a scoop to collect blood from a finger prick. Hem-Col LiHep was used for the analyses of cholesterol, creatinine, HDL cholesterol, LDL cholesterol and triglycerides and Hem-Col EDTA for HbA1c analysis. To assess buffer evaporation, tube weight was measured using a R200D analytical balance with 0.001 g precision (Sartorius AG, Göttingen, Germany). Over a period of six months, five tubes stored at room temperature containing 150 μl buffer were weight biweekly.

### 2.3 Internal standard, glycerol-3-phosphate, G3P

Glycerol-3-phosphate (G3P, EliTech Clinical Systems, Sees, France), a phosphoric ester of glycerol, was used as internal standard to calculate analyte concentrations as explained below. G3P was added in a standard amount (50 μl, 5 mmol/l) to the Li-Hep Hem-Col microtube before measurement of the Diabetes Care parameters and measured together with the other analytes. The dilution factor of the plasma and the concentration of the analytes could be calculated as described by Gootjes et al. [8]. Total precision of the assay was determined by measuring control samples twice a day for 20 days. Linearity was determined with a dilution range between 0 and 5 mmol/l G3P (0.0, 0.63, 1.25, 2.50, 3.75 and 5.0 mmol/l). The limit of quantitation (LoQ) was analysed by measuring different concentrations of G3P, ranging from 0 to 0.25 mmol/l (0.05, 0.10, 0.15 and 0.25 mmol/l).

200 random anonymized EDTA-plasma samples obtained by venepuncture were analysed for the presence of G3P after centrifugation.

### 2.4 Analysis

The analytes cholesterol, creatinine, HDL cholesterol, LDL cholesterol and triglycerides as well as G3P were measured in plasma on the Dimension Vista 500, using accompanying reagents (Siemens Healthcare Diagnostics, Tarrytown, USA). For G3P, reagents from EliTech Clinical Systems were used. HbA1c was measured on the TOSOH G8, with accompanying reagents (Tosoh Bioscience, Tokyo, Japan).

Hem-Col samples (n = 40) were centrifuged (Li-Hep) and directly analysed after 2 and 120 hours of storage at room temperature (approximately 21° Celsius) for stability experiments. Venous samples were centrifuged and directly analysed 2 hours after collection to simulate routine analysis. To assess the influence of temperature on the analytes, samples (n = 10) were stored for 120 hours at 4 or 37° Celsius before centrifugation and subsequent analysis.

The recovery of the plasma parameters was calculated with the following formulas:

A: Collected plasma in μl = (total volume buffer in μl * [G3P] buffer in mmol/l / measured [G3P] in mmol/l) – total buffer volume in μl
B: Dilution factor plasma = (200 ​μl / A) +1
  *Where 200 μl *indicates* 150 μl *buffer and* 50 μl* G3P standard per sample*

The recovery can thus be calculated as follows:

C: Recovery (%) = (concentration analyte in Hem-Col * B / concentration analyte in plasma) * 100

HbA1c is expressed as a proportion of the total haemoglobin concentration, therefore the results are independent of blood volume in the sample and recovery does not need to be calculated with the above-stated formulas. In regular measurement (venous material), whole blood is diluted 100 times by the TOSOH G8 (Tosoh Bioscience, Tokyo, Japan). To standardize Hem-Col measurements, the packed red blood cell fraction was diluted 200 times with wash buffer (Tosoh Bioscience, Tokyo, Japan) to a similar red blood cell concentration as whole blood used in routine measurements.

#### 2.4.1 Sensitivity analysis

LOQ was determined for cholesterol, creatinine, HDL cholesterol, LDL cholesterol and triglycerides. Random plasma was pooled and diluted with Hem-Col buffer in dilutions of 1–32 times. Analytes were measured 10 times per dilution step. The LoQ was determined for all five analytes as the concentration at which a CV of 10% was achieved.

### 2.5 Statistical analysis

Data were analysed using GraphPad Prism 5 (GraphPad Software, La Jolla, CA, USA), NCSS Statistical Software 12 (NCSS, Kaysville, Utah, USA), Microsoft Excel 2010 (Microsoft, Redmond, Washington, US) and MedCalc 18.11 (MedCalc Software, Ostend, Belgium). To assess potential differences, tube weight was analysed with paired T-testing at indicated time points (Graphpad). Sensitivity analysis was performed with Deming regression analysis (Graphpad). Method comparisons were performed using Bland-Altman analysis (MedCalc) as described by Altman et al. and Thienpont et al. [9,10]. Outliers within the experiments shown in Fig. 3, Fig. 4 were identified using Grubbs’ analysis and Rosner’s conclude procedure (MedCalc).

**Fig. 2:**
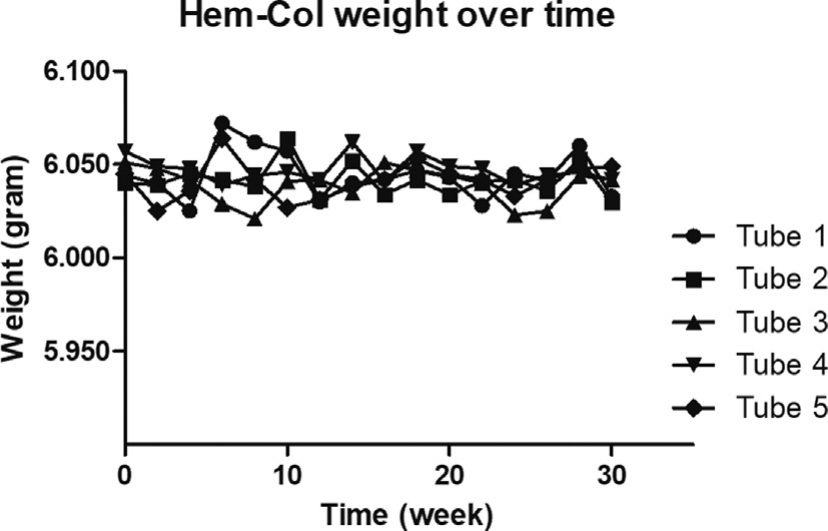
**Stability of the amount of buffer in the Hem-Col Device**.

**Fig. 3:**
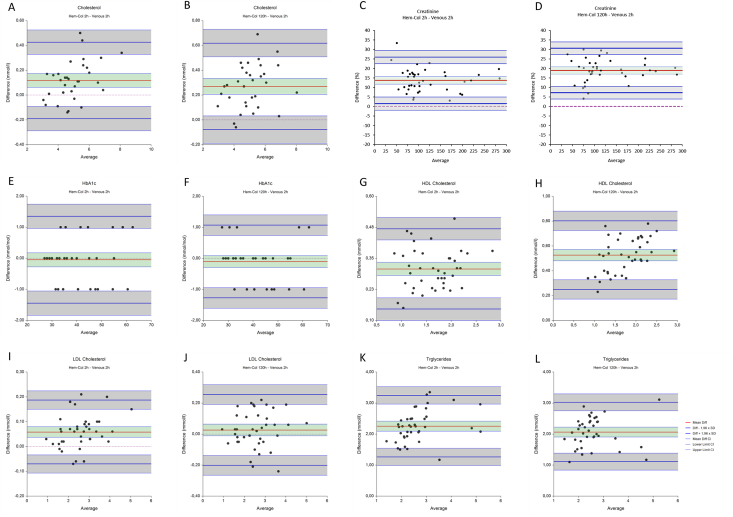
**Comparison of venous and Hem-col material after 2 and 120 hours of storage at room temperature.** The mean difference is indicated by a red line, in green the confidence interval (95% CI) is shown. Blue lines indicate the lower and upper limits of agreement (LoA, Diff – and + 1.96 xSD), in grey the confidence interval (95% CI) are shown. The dotted line indicates y ​= ​0.

**Fig. 4:**
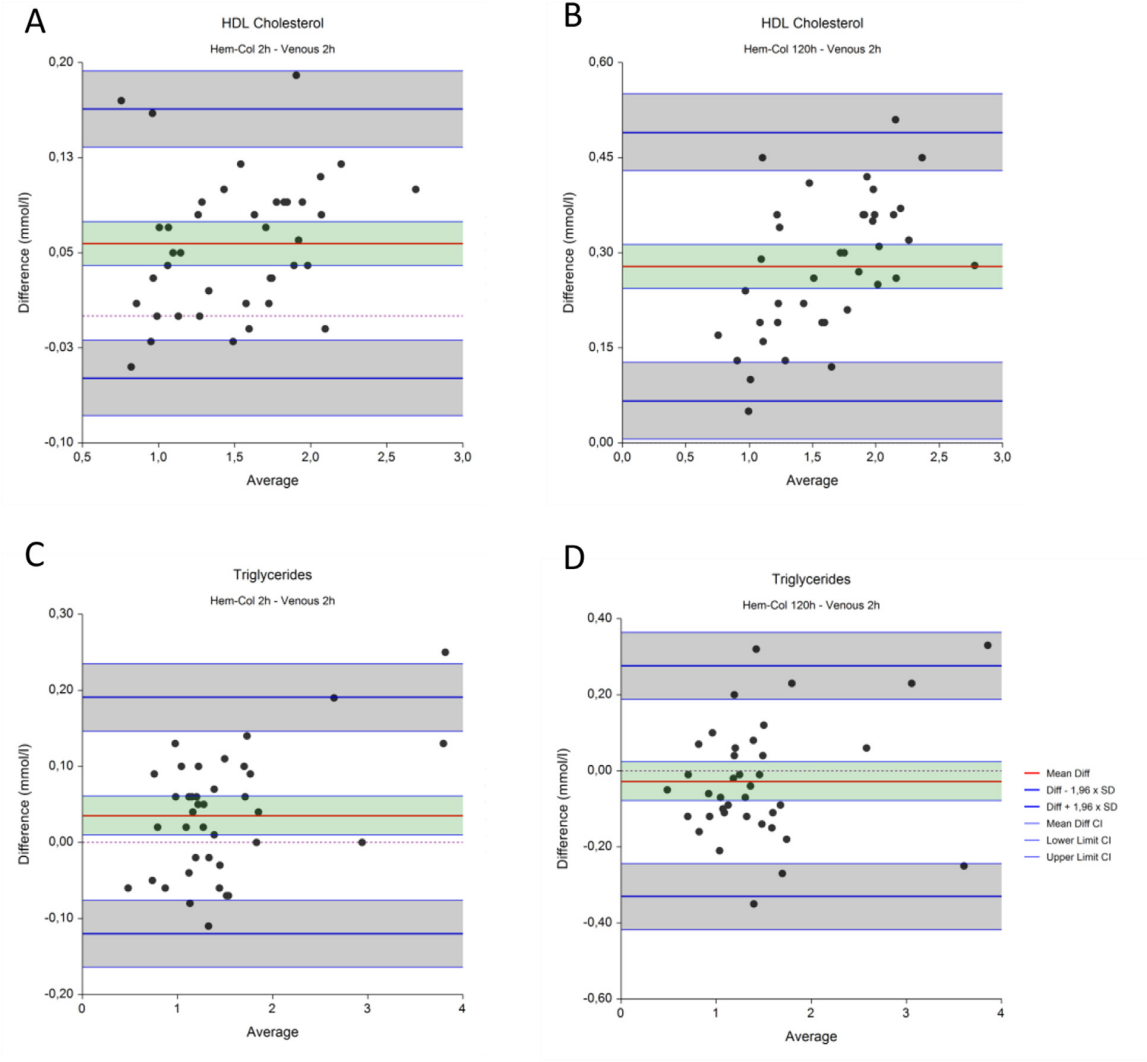
**Recalculated comparison of venous and Hem-col material after 2 and 120 hours of storage at room temperature.** The mean difference is indicated by a red line, in green the confidence interval (95% CI) is shown. Blue lines indicate the lower and upper limits of agreement (LoA, Diff – and + 1.96 xSD), in grey the confidence interval (95% CI) are shown. The dotted line indicates y ​= ​0.

## 3 Results

### 3.1 Buffer evaporation

Since the calculation of the recovery of analytes is dependent on the amount of buffer in the tube, it was assessed whether the amount of buffer was constant over a period of six months. Results are shown in Fig. 2. At week 10, 20 and 30, no significant difference in weight was observed, indicating no significant buffer evaporation over time.

### 3.2 G3P internal standard evaluation and recovery calculation

The details and performance of the G3P assay were previously described by Gootjes et al. [8]. Here, precision, linearity and analytical sensitivity were analysed. Total precision resulted in a variation coefficient of < 1%. Linearity was perfect (r^2^ = 1.0). The LoQ of 0.011 mmol/l with a coefficient of variation of 8.7%.

Hereafter, 200 random anonymous EDTA-plasma samples were analysed for the presence of G3P after centrifugation. G3P was undetectable in all samples (data not shown), indicating that G3P is not present in human plasma.

### 3.3 Sensitivity of analyte measurements in the low range

Because the capillary blood is diluted with the anticoagulant containing buffer in the Hem-Col, the analyte concentration is lower than in plasma collected via regular venepuncture. Therefore, performance of the different assays (cholesterol, creatinine, HDL cholesterol, LDL cholesterol and triglycerides) was assessed at relevant concentrations corresponding to expected concentrations of the Hem-Col material, which are approximately a factor three lower than the reference range. Results are shown in Table 1. The LoQ is below the expected concentration range of the samples for all analytes. HbA1c sensitivity analysis in the low range was not performed, since measuring HbA1c in the manual mode with Hem-Col material results in a similar concentration as in the regular measuring mode with venous blood.

**Table 1:** **Low concentration measurements of the different analytes**.

Analyte	Cholesterol	Creatinine	HDL	LDL	Triglycerides
Hem-Col	Li-Hep	Li-Hep	Li-Hep	Li-Hep	Li-Hep
Expected range	0–3 mmol/l	20–40 μmol/l	0.3–1 mmol/l	0.5–1.5 mmol/l	0.5–1 mmol/l
Linearity, r^2^	0.999	0.999	0.998	0.999	0.999
LoQ (CV 10%)	0.12 mmol/l	14.4 μmol/l	0.1 mmol/l	0.21 mmol/l	0.2 mmol/l

### 3.4 Comparison of Hem-Col and venepuncture measurements and stability over time

Each analyte measured in Hem-Col material was measured 2 and 120 hours after collection, and compared to venous material from the same patient stored for 2 hours, as shown in Fig. 3. Mean differences and limits of agreement (LoA) with accompanying 95% confidence intervals (CI) are summarized in Table 2. Acceptance limits were defined based on total allowable error and are also reported in Table 2 [11]. Cholesterol after two hours (Fig. 3A) shows a mean difference of 0.12 mmol/l, and limits of agreement of −0.19 and 0.43 mmol/l. The 95% confidence interval of the upper limit of agreement just exceeds the predefined acceptance limit of 0.50 mmol/l. After 120 hours (Fig. 3B), the mean difference increases to 0.27 with limits of agreement of −0.08 and 0.62. The upper limit of agreement is greater than the predefined acceptance limit of  ±0.50 mmol/l. The difference plots of creatinine (Fig. 3C and D) are shown with a percent y-scale, as the standard deviation of the differences of creatinine increases with concentration [12]. Creatinine after two hours (Fig. 3C) shows a mean difference of 13.66%, and limits of agreement of 1.32 mmol/l and 26.00 mmol/l. The 95% confidence intervals of both the mean difference and upper limit of agreement exceed the predefined acceptance limit of 15%. After 120 hours (Fig. 3D), the mean difference increases to 18.95 with limits of agreement of −7.24% and 30.65%. The upper limit of agreement is greater than the predefined acceptance limit of  ±15%. HbA1c (Fig. 3E) after two hours shows a mean difference of −0.05 mmol/mol, and limits of agreement of −1.45 and 1.35 mmol/l. All intervals are within the predefined acceptance limit of  ±9%. After 120 hours (Fig. 3F), no change in acceptance of mean difference or limits of agreement is observed. HDL cholesterol (Fig. 3G) after two hours results in a mean difference of 0.31 mmol/l, and limits of agreement of 0.15 and 0.47 mmol/l. All intervals exceed the predefined acceptance limit of  ±0.20 mmol/l. After 120 hours (Fig. 3H), the mean difference increases to 0.53 mmol/l, and the limits of agreement increase to 0.25 and 0.80 mmol/l. These differences further exceed the acceptance limit of 0.20 mmol/L. Also, the mean difference increases with increasing HDL concentration after 120 hours. LDL cholesterol (Fig. 3I) after two hours has a mean difference of 0.06 mmol/l, and limits of agreement of −0.07 and 0.19 mmol/l. All intervals fall well within the predefined acceptance limit of  ±0.39 mmol/l. After 120 hours (Fig. 3J) the mean difference decreases to 0.03, with limits of agreement of −0.20 and 0.25. These intervals still fall within the predefined acceptance limit of  ± 0.39 mmol/l. Triglycerides (Fig. 3K) after two hours show a mean difference of 2.26 mmol/l, and limits of agreement of 1.26 and 3.25 mmol/l. All intervals far exceed the predefined acceptance limit of  ±25%. After 120 hours (Fig. 3K) the mean difference decreases to 2.07, with limits of agreement of 1.11 and 3.02. These intervals also exceed the predefined acceptance limit of  ±25%.

**Table 2:** **Comparison of analytes measured in venous material after 2 hours at room temperature and Hem-Col material after storage for 2 hours and 120 hours.** Mean differences of Hem-Col minus venous results are shown, together with the lower and upper limits of agreements (LoA) with accompanying confidence intervals (CI).

		Mean Difference (CI)	Lower LoA (CI)	Upper LoA (CI)	Acceptance limits
Cholesterol (mmol/l)	2 h	0.12 (0.06–0.17)	−0.19 (−0.29 - -0.09)	0.43 (0.33–0.52)	0.50^1^
	120 h	0.27 (0.21–0.33)	−0.08 (−0.19 - -0.03)	0.62 (0.51–0.73)	0.50^1^
Creatinine (%)	2 h	13.66 (11.62–15.70)	1.32 (−2.20 - 4.84)	26.00 (22.48–29.52)	15%^2^
	120 h	18.95 (17.04–20.85)	7.24 (3.95–10.53)	30.65 (27.36–33.94)	15%^2^
HbA1c (mmol/mol)	2 h	−0.05 (−0.28 - 0.18)	−1.45 (−1.84 - -1.06)	1.35 (0.96–1.74)	9%^3^
	120 h	−0.10 (−0.30 - 0.09)	−1.27 (−1.61 - -0.94)	1.07 (0.74–1.40)	9%^3^
HDL Cholesterol (mmol/l)	2 h	0.31 (0.28–0.33)	0.15 (0.10–0.19)	0.47 (0.42–0.51)	0.20^1^
	120 h	0.53 (0.48–0.57)	0.25 (0.17–0.33)	0.80 (0.72–0.88)	0.20^1^
LDL Cholesterol (mmol/l)	2 h	0.06 (0.04–0.08)	−0.07 (−0.11 - -0.03)	0.19 (0.15–0.22)	0.39^4^
	120 h	0.03 (−0.01 - 0.06)	−0.20 (−0.27 - -0.14)	0.25 (0.19–0.32)	0.39^4^
Triglycerides (mmol/l)	2 h	2.26 (2.09–2.42)	1.26 (0.98–1.54)	3.25 (2.97–3.53)	25%^4^
	120 h	2.07 (1.91–2.22)	1.11 (0.84–1.38)	3.02 (2.75–3.29)	25%^4^

Because creatinine, HDL cholesterol and triglycerides show mean differences that exceed the acceptance limits, we tested Hem-Col buffer after adding G3P for the presence of these analytes. Creatinine was undetectable in Hem-Col buffer. However, HDL cholesterol and triglycerides did give a constant measurement signal when measured in Hem-Col buffer (data not shown). This allowed for recalculation of the data using the following formula to account not only for the dilution of plasma, but also for the dilution of the signal of the analytes in undiluted Hem-Col buffer:

Analyte concentration: (measured [HDL cholesterol or triglycerides] in Hem-Col sample in mmol/l) * (buffer volume in μl ​+ ​collected plasma in μl)) – ([HDL cholesterol or triglycerides] in plain buffer in mmol/l * volume buffer in μl)) / collected plasma in μl.

The results are shown in Fig. 4 and Table 3. After recalculation, HDL cholesterol (Fig. 4A) after two hours shows a mean difference of 0.06 mmol/l, and limits of agreement of 0.05 and 0.16 mmol/l. These intervals fall within the predefined acceptance limit of  ±0.20 mmol/l. After 120 hours (Fig. 4B) the mean difference increases to 0.28, with limits of agreement of 0.07 and 0.49, exceeding the acceptance limit of  ±0.20 mmol/l. After recalculation, triglycerides (Fig. 4C) after two hours result in a mean difference of 0.04 mmol/l, and limits of agreement of −0.12 and 0.19 mmol/l. These intervals fall within the predefined acceptance limit of  ±25%. After 120 hours (Fig. 4D) the mean difference decreases to 2.07, with limits of agreement of −0.33 and 0.28 mmol/l, just exceeding the acceptance limit of  ±25%.

**Table 3:** **Recalculated HDL cholesterol and triglycerides comparison in venous material after 2 hours at room temperature and Hem-Col material after storage for 2 hours and 120 hours.** Mean differences of Hem-Col minus venous results are shown, together with the lower and upper limits of agreements (LoA) with accompanying confidence intervals (CI).

		Mean Difference (CI)	Lower LoA (CI)	Upper LoA (CI)	Acceptance limits
HDL Cholesterol (mmol/l)	2 h	0.06 (0.04–0.07)	−0.05 (−0.08 - -0.02)	0.16 (0.13–0.19)	0.20
	120 h	0.28 (0.24–0.31)	0.07 (0.01–0.13)	0.49 (0.43–0.55)	0.20
Triglycerides (mmol/l)	2 h	0.04 (0.01–0.06)	−0.12 (−0.16 - -0.08)	0.19 (0.15–0.24)	25%
	120 h	−0.03 (−0.08 - 0.02)	−0.33 (−0.42 - -0.24)	0.28 (0.19–0.36)	25%

### 3.5 Stability at different temperatures

**Fig. 5:**
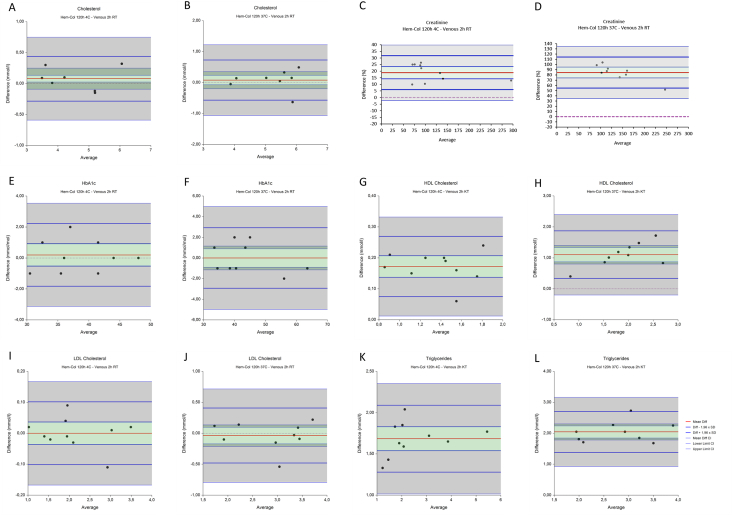
**Comparison of venous (2 hours, RT) and Hem-col material (120 hours) at different temperatures.** The mean difference is indicated by a red line, in green the confidence interval (95% CI) is shown. Blue lines indicate the lower and upper limits of agreement (LoA, Diff – and + 1.96 xSD), in grey the confidence interval (95% CI) are shown. The dotted line indicates y ​= ​0.

Temperature sensitivity was assessed after 120 hours of storage at 4 and 37 °C and compared to venous material stored for 2 hours at room temperature (Fig. 5 and Table 4). Since sample size is small (n = 10), the 95% CI’s are relatively large. Cholesterol stored at 4 °C (Fig. 5A and B) has a mean difference of 0.08 mmol/l, and limits of agreement of −0.28 and 0.44 mmol/l. Storage at 37 °C results in a mean difference of 0.08, and limits of agreement of −0.57 and 0.73 mmol/l. The 95% confidence intervals of the lower and upper limit of agreement at both temperatures exceed the predefined acceptance limit of 0.50 mmol/l. Creatinine (Fig. 5C) shows a mean difference of 18.88% after storage at 4 °C, and limits of agreement of 6.05 and 31.70%. The 95% confidence intervals of both the mean difference and upper limit of agreement exceed the predefined acceptance limit of 15%. After storage at 37 °C (Fig. 5D), the mean difference increases to 84.64% with limits of agreement of 54.94 and 114.33%. The mean difference and limits of agreement is far greater than the predefined acceptance limit of  ±15%. HbA1c (Fig. 5E) at 4 °C shows a mean difference of 0.20 mmol/mol, and limits of agreement of −1.82 and 2.22 mmol/l. All intervals are within the predefined acceptance limit of  ± 9%. After 120 hours (Fig. 5F), no change in mean difference or limits of agreement is observed. HDL cholesterol (Fig. 5G) storage at 4 °C results in a mean difference of 0.17 mmol/l, and limits of agreement of 0.08 and 0.27 mmol/l. All intervals exceed the predefined acceptance limit of  ±0.20 mmol/l. Storage at 37 °C (Fig. 5H) results in an increase of the mean difference to 1.10 mmol/l, and the limits of agreement increase to 0.34 and 1.87 mmol/l. These differences further exceed the acceptance limit of 0.20 mmol/L. LDL cholesterol (Fig. 5I) storage at 4 °C shows no mean difference (0.00 mmol/l, 95% CI: ±0.04), and limits of agreement of −0.10 and 0.10 mmol/l. All intervals are within the predefined acceptance limit of ± 0.39 mmol/l. Storage at 37 °C (Fig. 3J) gives a mean difference of −0.04, with limits of agreement of −0.48 and 0.41. These LoA exceed the predefined acceptance limit of  ± 0.39 mmol/l. Triglycerides (Fig. 5K-L) storage at 4 °C show a mean difference of 1.68 mmol/l, and limits of agreement of 1.28 and 2.09 mmol/l. All intervals far exceed the predefined acceptance limit of  ± 25%. Storage at 37 °C (Fig. 3K) gives a mean difference of 2.04 mmol/l, with limits of agreement of 1.39 and 2.70 mmol/l. These intervals also exceed the predefined acceptance limit of  ± 25%.

**Table 4:** **Comparison of analytes measured in venous material after 2 hours at room temperature and Hem-Col material after 120 hours at different temperatures.** Mean differences of Hem-Col minus venous results are shown, together with the lower and upper limits of agreements (LoA) with accompanying confidence intervals (CI).

		Mean Difference (CI)	Lower LoA (CI)	Upper LoA (CI)	Acceptance limits
Cholesterol (mmol/l)	4 °C	0.08 (−0.09 - 0.25)	−0.28 (−0.59 - 0.02)	0.44 (0.13–0.75)	0.50
	37 °C	0.08 (−0.20 - 0.36)	−0.57 (−1.06 - -0.08)	0.73 (0.23–1.22)	0.50
Creatinine (%)	4 °C	18.88 (14.19–23.56)	6.05 (−2.24 - 14.34)	31.70 (23.41–39.99)	15%
	37 °C	84.64 (72.99–96.28)	54.94 (34.24–75.65)	114.33 (93.63–135.04)	15%
HbA1c (mmol/mol)	4 °C	0.20 (−0.54 - 0.94)	−1.82 (−3.13 - 0.52)	2.22 (0.92–3.53)	9%
	37 °C	0.00 (−1.15 - 1.15)	−2.94 (−4.99 - -0.89)	2.94 (0.89–4.99)	9%
HDL Cholesterol (mmol/l)	4 °C	0.17 (0.14–0.21)	0.08 (0.01–0.14)	0.27 (0.21–0.33)	0.20
	37 °C	1.10 (0.80–1.40)	0.34 (−0.20 - 0.87)	1.87 (1.33–2.40)	0.20
LDL Cholesterol (mmol/l)	4 °C	0.00 (−0.04 - 0.04)	−0.10 (−0.17 - -0.04)	0.10 (0.04–0.17)	0.39
	37 °C	−0.04 (−0.21 - 0.14)	−0.48 (−0.79 - -0.17)	0.41 (0.10–0.72)	0.39
Triglycerides (mmol/l)	4 °C	1.68 (1.54–1.83)	1.28 (1.01–1.54)	2.09 (1.83–2.35)	25%
	37 °C	2.04 (1.79–2.30)	1.39 (0.93–1.85)	2.70 (2.24–3.16)	25%

**Fig. 6:**
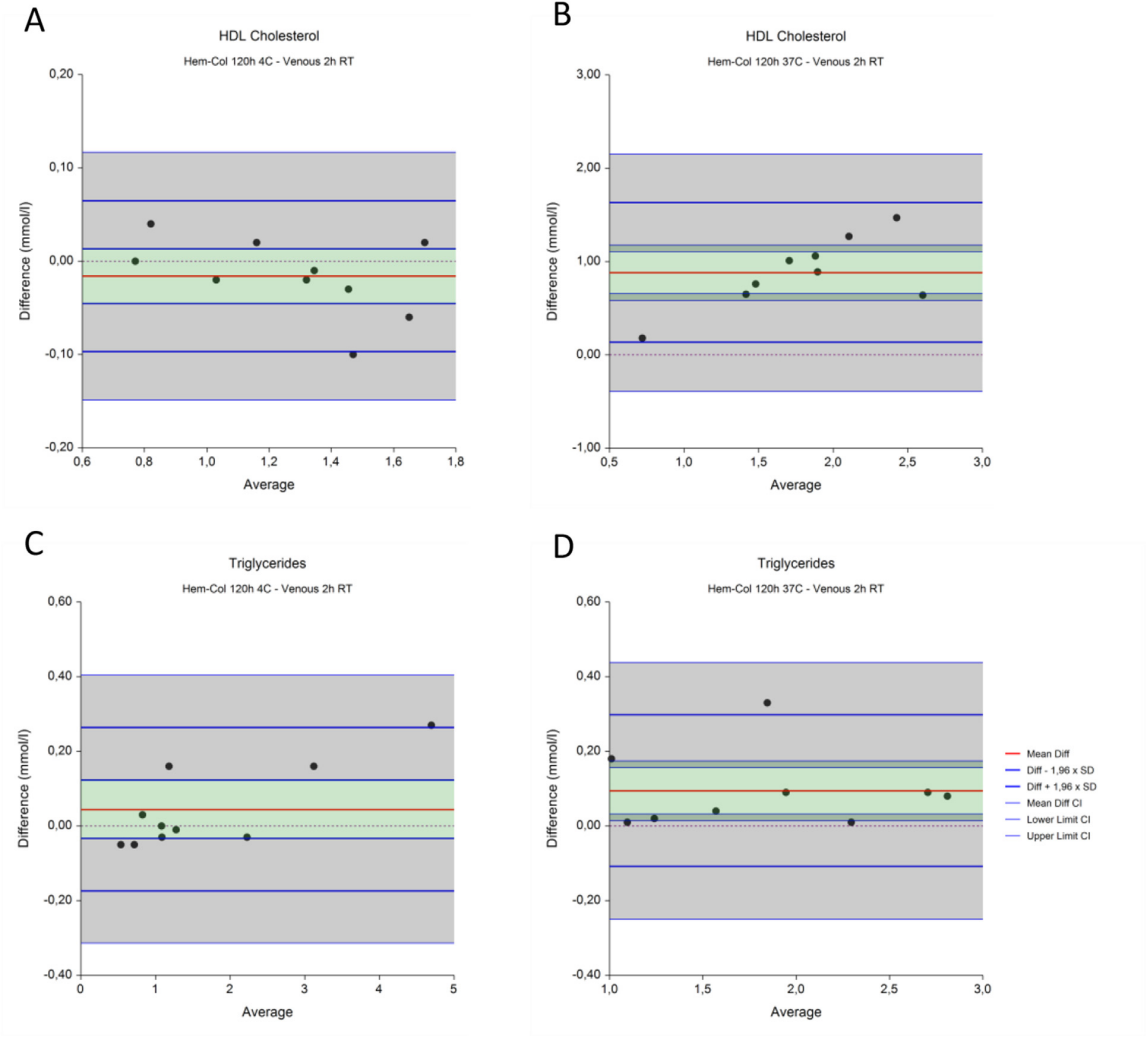
**Recalculated comparison of venous (2 hours, RT) and Hem-col material (120 hours) at different temperatures.** The mean difference is indicated by a red line, in green the confidence interval (95% CI) is shown. Blue lines indicate the lower and upper limits of agreement (LoA, Diff – and + 1.96 xSD), in grey the confidence interval (95% CI) are shown. The dotted line indicates y ​= ​0.

HDL cholesterol and triglycerides recalculated as described above are shown in Fig. 6 and Table 5. After recalculation, HDL cholesterol (Fig. 6A) at 4 °C shows a mean difference of −0.02 mmol/l, and limits of agreement of −0.10 and 0.06 mmol/l. These intervals are within the predefined acceptance limit of  ±0.20 mmol/l. At 37 °C (Fig. 6B) the mean difference increases to 0.88, with limits of agreement of 0.13 and 1.63, exceeding the acceptance limit of  ±0.20 mmol/l. After recalculation, triglycerides (Fig. 6C) at 4 °C shows a mean difference of 0.05 mmol/l, and limits of agreement of −0.17 and 0.26 mmol/l. At 37 °C (Fig. 6D) the mean difference is 0.09, with limits of agreement of −0.11 and 0.30 mmol/l. The mean differences and intervals at both temperatures are within the predefined acceptance limit of  ±25%.

**Table 5:** **Recalculated HDL cholesterol and triglycerides comparison in venous material after 2 hours at room temperature and Hem-Col material after 120 hours at different temperatures.** Mean differences of Hem-Col minus venous results are shown, together with the lower and upper limits of agreements (LoA) with accompanying confidence intervals (CI).

		Mean Difference (CI)	Lower LoA (CI)	Upper LoA (CI)	Acceptance limits
HDL Cholesterol (mmol/l)	4 °C	−0.02 (−0.05 - 0.01)	−0.10 (−0.15 - -0.04)	0.06 (0.01–0.12)	0.20
	37 °C	0.88 (0.59–1.17)	0.13 (−0.39 - 0.65)	1.63 (1.11–2.15)	0.20
Triglycerides (mmol/l)	4 °C	0.05 (−0.03 - 0.12)	−0.17 (−0.31 - -0.03)	0.26 (0.12–0.40)	25%
	37 °C	0.09 (0.01–0.17)	−0.11 (−0.25 - 0.03)	0.30 (0.16–0.44)	25%

## 4 Discussion and conclusion

This is the first study to report on the agreement of the measurement of the Diabetes Care parameters cholesterol, creatinine, HbA1c, HDL cholesterol, LDL cholesterol and triglycerides between blood collected via the novel Hem-Col device and by regular venepuncture.

When searching the term “finger prick” in PubMed, an increasing number of publications is noticeable, which reflects a growing interest in this blood collection method. Blood collection by finger prick has advantages over drawing blood by venepuncture, for both patients and healthcare providers, yet most often blood is still collected via venepuncture. For example, it can be collected by patients themselves and only a small sample volume is required. A major bottleneck for laboratories is the short timeframe of several hours in which the cells need to be separated from the plasma to ensure valid results. The development of methods to keep analytes stable in whole blood for longer periods of time, would be an important stimulant for the widespread use of blood collection via finger prick. Hem-Col is a device designed for this purpose.

Hem-Col tubes and buffer are designed to preserve blood collected by finger prick in order to allow for reliable measurement of clinical chemistry analytes after days of storage before separating cells from plasma. Since the amount of capillary blood collected by finger prick varies considerably, the dilution of the buffer with blood must be calculated. To this end, an internal standard of known concentration can be added to the dilution buffer. The ideal internal standard is simple to measure with high precision, absent from blood and stable over time. In the past, G3P has been described as internal standard [8]. We show that G3P can be easily measured with high precision, and found G3P to be absent from blood, but, as reported before, G3P is not stable in blood due to hydrolysis by the plasma enzyme alkaline phosphatase [13]. To avoid degradation, G3P was added shortly before analysis. Since an extra handling in the pre-analytical process is not preferable, we advise to consider an alternative internal standard that can be added to the Hem-Col buffer before blood taking. For example, choline can be considered [13]. Loss of Hem-Col buffer by evaporation, which would lead to erroneous calculation of the plasma dilution factor, is a risk that was assessed by a weighing experiment. Over a period of thirty weeks, no decrease in weight of Hem-Col tubes was noticed, indicating proper closing of the lid. Since the collected capillary blood is diluted with the Hem-Col buffer, the analytes are measured in a lower range than they would be in undiluted plasma. We show that the Diabetes Care parameters have acceptable limits of quantitation when measured at low concentrations in Hem-Col buffer.

For Hem-Col to be a suitable alternative to venepuncture, the agreement between analytes measured in material collected via Hem-Col and by venepuncture must be acceptable. To judge the agreement, we determined whether the 95% confidence intervals of the mean difference and limits of agreement were equal to or smaller than predefined acceptance limits. These acceptance limits were based on proficiency testing criteria for acceptable analytical performance [11]. Other factors such as user-friendliness, costs and implementation, are also important to take into consideration when determining if one method can replace another, but these aspects were beyond the scope of this study.

Because time and temperature are of influence on laboratory results, the effects of these variables were assessed in this study. HbA1c, for which dilution and thereby the internal G3P standard are not relevant, shows excellent agreement between Hem-Col and venepuncture after storage for 120 hours at room temperature, and no critical effect of storage at 4 and 37 °C was observed. LDL cholesterol is also stable in Hem-Col for up to 120 hours at 4 °C and room temperature, but at 37 °C the differences seem to increase, while the mean difference remains unchanged. Creatinine shows a strong positive bias, which is both time and temperature dependent. The mean difference is increased after 120 hours at room temperature and is strongly increased after 120 hours at 37 °C, while it seems to remain constant at 4 °C. Without adapting the reference range, result cannot be reported as such. However, the spread around the mean difference is acceptable for all tested times and temperatures except 37 °C, and if the mean difference is predictable, it would be possible to correct for this discrepancy by a proportional correction factor to obtain acceptable agreement. Cholesterol shows a small positive bias with an acceptable variation around the mean difference at all tested storage times and temperatures. When eliminating this bias with a correction factor, acceptable agreement can be achieved. Since mean differences do vary between the tested conditions, consistency in handling the material is desirable. Both HDL cholesterol and triglycerides have such a large bias that agreement is unacceptable. These differences are primarily explained by the fact that both analytes are measurable in undiluted Hem-col buffer. The HDL cholesterol signal can be explained by interference of G3P with the HDL cholesterol measurement, as G3P is part of the chemical reaction equation of HDL cholesterol measurement. A plausible explanation for the creatinine signal was not identified. Since this interference is predictable, the dilution factor should be calculated with a different formula, which results in acceptable agreement for triglycerides. HDL cholesterol agreement is acceptable at room temperature for 2 hours and 4 °C for 120 hours. Because of a time and temperature dependency, a correction factor must be included to obtain acceptable agreement after 120 hours of storage.

Further studies will be initiated to confirm current results and to investigate other important factors apart from the technical and agreement results within this report, required to determine whether blood collection via Hem-Col can replace the venepuncture for Diabetes Care parameters. Furthermore, it would be of interest to evaluate the potential increase in patient commitment because of streamlining and simplifying disease management, and thereby investigate whether this possibly reduces health care costs.

In conclusion, the results of this study show that Hem-Col is suitable for the measurement of HbA1c after storage for up to 120 hours at temperatures ranging from 4 to 37 °C. Cholesterol, creatinine, HDL cholesterol, LDL cholesterol and triglycerides can be measured after 120 hours of storage in Hem-Col buffer, if high temperatures, possibly above room temperature, can be avoided. Also, laboratories should feel comfortable using correction factors, or, alternatively, make adaptations to reported reference intervals. When taking these considerations into account, Hem-Col can be a potential alternative for current venous or capillary blood collections for Diabetes Care parameters.

## Declaration of interest

FV, RC and HV hold an equity interest in Labonovum B.V. and serve in an uncompensated advisory/consulting role for Labonovum B.V.. MH declares no conflict of interest.

## Role of the funding source

Hem-Col devices and technical support were provided by Labonovum B.V.
